# Supplementary material for: Assessment of Various Density Functional Theory Methods for Finding Accurate Structures of Actinide Complexes
Source: Molecules. 2022 Feb 23;27(5):1500. doi: 10.3390/molecules27051500 (PMC8911565; doi:10.3390/molecules27051500)
Supplement: Supplementary file 1 [file molecules-27-01500-s001.zip › molecules-1576012-supplementary.pdf]

Table s1. Average bond length of U-F bonds in the optimized structure with 6-31G(d) and with 6-31+G(d) [23].

**DFT Method combination**		Average Length (Å)	Deviation (Å)
Experiment[23]		1.996	-
6-31G(d)	B3LYP	2.007	0.011
	BLYP	2.032	0.036
	BP86	2.018	0.022
	BPW91	2.019	0.023
	PBE	2.016	0.020
	N12	1.996	0.000
	M06L	2.020	0.024
	M11L	1.974	0.022
	MN12L	1.986	0.010
	TPSS	2.014	0.018
	B3P86	1.994	0.002
	B3PW91	1.997	0.001
	PBE0	1.990	0.006
	M06	1.995	0.001
	TPSSh	2.004	0.008
	wB97X	1.994	0.002
	M11	1.998	0.002
	N12SX	1.990	0.006
	MN12SX	1.986	0.010
6-31G+(d)	B3LYP	2.038	0.042
	BLYP	2.038	0.042
	BP86	2.022	0.026
	BPW91	2.022	0.026
	PBE	2.021	0.025
	N12	2.001	0.005
	M06L	2.021	0.025
	M11L	1.972	0.024
	MN12L	1.987	0.009
	TPSS	2.018	0.022
	B3P86	1.996	0.000
	B3PW91	1.999	0.003
	PBE0	1.992	0.004
	M06	1.998	0.002

	TPSSh	2.006	0.010
	wB97X	1.997	0.001
	M11	2.002	0.006
	N12SX	1.993	0.003
	MN12SX	1.988	0.008

Table s2. Average bond length of Am-Cl bonds in the optimized structure with 6-31G(d) and with 6-31+G(d) [9].

**DFT Method combination**		Average Length (Å)	Deviation (Å)
Experiment[9]		2.724	-
6-31G(d)	B3LYP	2.834	0.110
	BLYP	2.858	0.134
	BP86	2.818	0.094
	BPW91	2.826	0.102
	PBE	2.815	0.091
	N12	2.792	0.068
	M06L	2.830	0.106
	M11L	2.807	0.083
	MN12L	2.786	0.062
	TPSS	2.814	0.090
	B3P86	2.799	0.075
	B3PW91	2.811	0.087
	PBE0	-	-
	M06	2.809	0.085
	TPSSh	2.809	0.085
	wB97X	2.815	0.091
	M11	2.815	0.091
	N12SX	2.797	0.073
6-31G+(d)	MN12SX	2.804	0.080
	B3LYP	2.837	0.113
	BLYP	2.874	0.150
	BP86	2.846	0.122
	BPW91	2.842	0.118
	PBE	2.832	0.108
	N12	2.807	0.083
	M06L	2.846	0.122

	M11L	2.825	0.102
	MN12L	2.805	0.081
	TPSS	2.831	0.107
	B3P86	2.813	0.089
	B3PW91	2.825	0.101
	PBE0	-	-
	M06	2.820	0.096
	TPSSh	2.824	0.100
	wB97X	2.826	0.102

Table S3. (**a**) Bond length and (**b**) Angle of UO_2(L)Alc. in the optimized structure [17].

a

Row	Symbol	Bond Length				Deviation		
		B3P86	B3PW91	M06	Experiment [17]	B3P86	B3PW91	M06
1	O							
2	C	1.302	1.303	1.298	1.330	0.028	0.028	0.032
3	C	1.438	1.439	1.437	1.422	0.016	0.017	0.015
4	C	1.388	1.390	1.389	1.399	0.011	0.009	0.010
5	C	1.363	1.365	1.362	1.385	0.021	0.020	0.023
6	H	1.085	1.086	1.088	0.949	0.136	0.136	0.138
7	C	1.411	1.413	1.410	1.406	0.005	0.007	0.004
8	H	1.088	1.089	1.091	0.950	0.138	0.138	0.140
9	C	1.384	1.385	1.381	1.379	0.005	0.006	0.003
10	H	1.085	1.086	1.089	0.949	0.136	0.136	0.139
11	C	4.854	4.915	4.658	5.108	0.254	0.193	0.450
12	H	1.095	1.096	1.097	0.980	0.116	0.117	0.117
13	H	1.096	1.097	1.098	0.980	0.116	0.116	0.118
14	H	1.094	1.095	1.096	0.981	0.114	0.114	0.116
15	U	2.246	2.251	2.278	2.305	0.059	0.054	0.027
16	O	1.767	1.768	1.752	1.782	0.015	0.014	0.030
17	O	1.769	1.770	1.752	1.782	0.012	0.012	0.029
18	C	1.441	1.442	1.439	1.456	0.016	0.014	0.018
19	H	1.097	1.098	1.103	0.949	0.148	0.148	0.154

20	N	1.291	1.292	1.290	1.283	0.008	0.009	0.007
21	C	1.456	1.458	1.459	1.471	0.015	0.012	0.011
22	H	1.106	1.106	1.111	0.990	0.116	0.116	0.121
23	H	1.104	1.104	1.103	0.990	0.114	0.114	0.114
24	C	1.497	1.499	1.491	1.537	0.041	0.038	0.046
25	C	1.490	1.493	1.486	1.523	0.033	0.030	0.037
26	H	1.104	1.105	1.106	1.070	0.034	0.035	0.036
27	H	1.096	1.097	1.099	1.070	0.026	0.027	0.029
28	C	1.493	1.495	1.491	1.539	0.047	0.044	0.049
29	H	1.099	1.100	1.106	0.990	0.109	0.110	0.116
30	H	1.096	1.097	1.100	0.990	0.107	0.107	0.111
31	N	1.474	1.476	1.469	1.467	0.007	0.008	0.002
32	C	1.290	1.290	1.288	1.284	0.006	0.007	0.005
33	H	1.099	1.099	1.105	0.949	0.150	0.150	0.156
34	C	1.441	1.443	1.439	1.460	0.019	0.017	0.022
35	C	1.425	1.427	1.425	1.406	0.019	0.020	0.018
36	C	1.411	1.413	1.412	1.412	0.001	0.001	0.000
37	H	1.086	1.087	1.089	0.949	0.137	0.138	0.140
38	C	1.383	1.385	1.381	1.375	0.009	0.010	0.006
39	H	1.087	1.088	1.090	0.951	0.136	0.137	0.139
40	C	1.402	1.403	1.401	1.393	0.008	0.010	0.008
41	H	1.085	1.086	1.087	0.949	0.136	0.136	0.138
42	C	1.382	1.383	1.379	1.381	0.000	0.002	0.002
43	H	1.088	1.089	1.092	0.949	0.139	0.140	0.142
44	O	1.307	1.308	1.300	1.324	0.017	0.017	0.025
45	O	2.434	2.438	2.424	2.444	0.011	0.006	0.021
46	H	0.981	0.980	0.979	0.812	0.169	0.168	0.167
47	C	1.433	1.434	1.431	1.454	0.020	0.019	0.023
48	H	1.092	1.092	1.096	0.990	0.102	0.103	0.106
49	H	1.097	1.098	1.101	0.990	0.108	0.108	0.111
50	H	1.097	1.097	1.099	1.070	0.027	0.027	0.029

Average	1.386	1.389	1.383	1.346	0.066	0.064	0.071

b

Row	Symbol	Bond Angle				Deviation		
		B3P86	B3PW91	M06	Experiment [17]	B3P86	B3PW91	M06
1	O							
2	C							
3	C	120.937	120.955	121.543	120.910	0.027	0.045	0.633
4	C	116.493	116.501	116.737	119.673	3.181	3.172	2.937
5	C	127.443	127.445	127.297	121.636	5.807	5.809	5.661
6	H	123.389	123.364	123.398	120.956	2.434	2.409	2.443
7	C	114.998	114.999	115.052	117.987	2.989	2.989	2.935
8	H	119.069	119.081	119.182	119.117	0.048	0.037	0.065
9	C	121.683	121.677	121.657	121.800	0.118	0.123	0.143
10	H	120.823	120.770	120.872	119.828	0.995	0.943	1.045
11	C	119.286	120.766	114.114	121.569	2.283	0.803	7.455
12	H	115.805	116.462	114.533	152.512	36.707	36.050	37.979
13	H	124.810	124.394	127.518	95.086	29.724	29.308	32.432
14	H	88.689	88.399	86.807	71.771	16.918	16.627	15.036
15	U	131.622	132.155	128.795	130.322	1.300	1.833	1.527
16	O	93.867	93.676	94.508	87.267	6.600	6.408	7.241
17	O	87.256	87.413	86.330	90.962	3.706	3.549	4.632
18	C	122.901	122.931	122.910	120.654	2.247	2.278	2.257
19	H	114.599	114.504	114.523	116.640	2.042	2.137	2.117
20	N	126.882	126.965	126.790	126.687	0.195	0.278	0.103
21	C	116.786	116.718	117.296	117.108	0.321	0.390	0.189
22	H	110.706	110.750	109.782	108.869	1.837	1.881	0.913
23	H	106.681	106.661	107.385	108.834	2.153	2.173	1.449
24	C	114.406	114.443	113.199	113.533	0.873	0.909	0.334
25	C	117.122	117.069	119.541	107.313	9.808	9.756	12.227
26	H	111.727	111.812	111.285	109.471	2.255	2.340	1.814
27	H	111.734	111.720	111.848	109.471	2.263	2.249	2.377
28	C	119.910	119.833	120.302	110.875	9.035	8.957	9.426

29	H	110.116	110.094	110.871	108.954	1.161	1.139	1.917
30	H	111.143	111.047	111.349	109.012	2.131	2.035	2.337
31	N	110.346	110.567	108.709	112.800	2.454	2.234	4.091
32	C	116.886	116.866	117.225	116.712	0.173	0.154	0.512
33	H	117.783	117.802	117.590	117.120	0.663	0.682	0.470
34	C	127.841	127.886	128.404	125.859	1.982	2.027	2.546
35	C	122.690	122.676	123.040	121.452	1.238	1.224	1.587
36	C	117.751	117.730	117.463	118.107	0.356	0.377	0.644
37	H	117.863	117.901	117.557	119.542	1.679	1.641	1.985
38	C	121.239	121.262	121.423	120.842	0.397	0.419	0.580
39	H	119.244	119.249	119.220	119.297	0.053	0.048	0.077
40	C	121.054	121.044	121.124	121.475	0.421	0.430	0.351
41	H	120.647	120.648	120.725	120.868	0.221	0.220	0.143
42	C	118.665	118.668	118.477	118.370	0.295	0.298	0.107
43	H	119.941	119.881	119.979	119.183	0.758	0.698	0.796
44	O	121.688	121.697	122.223	121.415	0.273	0.282	0.808
45	O	31.790	31.146	34.492	19.091	12.699	12.055	15.401
46	H	95.336	94.857	96.300	83.752	11.584	11.104	12.548
47	C	35.459	35.433	35.548	33.732	1.727	1.701	1.816
48	H	104.950	105.017	105.000	108.894	3.944	3.877	3.894
49	H	109.633	109.686	109.595	108.997	0.636	0.689	0.597
50	H	111.646	111.618	111.916	109.471	2.175	2.146	2.444
Average		112.153	112.172	112.113	110.746	1.407	1.426	1.367
